# Acute cannabis intoxication in the emergency department: the effect of legalization

**DOI:** 10.1186/s12873-021-00428-0

**Published:** 2021-03-17

**Authors:** Robert Baraniecki, Puru Panchal, Danya Deepsee Malhotra, Alexandra Aliferis, Zaka Zia

**Affiliations:** 1 St. Joseph’s Healthcare Hamilton, Emergency Department, 50 Charlton Ave E, Hamilton, ON L8N 4A6 Canada; 2 Department of Family Medicine, Division of Emergency Medicine, McMaster University, Hamilton, ON Canada; 3 The Research Institute of St. Joe’s Hamilton, Hamilton, ON Canada; 4 Michael G. DeGroote School of Medicine, McMaster University, Hamilton, ON Canada

**Keywords:** Cannabis, Emergency, Toxicology, Health policy

## Abstract

**Background:**

On October 17, 2018, the Cannabis Act decriminalized the recreational use of cannabis in Canada. This study seeks to determine how legalization of cannabis has impacted emergency department (ED) visits for acute cannabis intoxication.

**Methods:**

We conducted a retrospective chart review at an academic ED in Hamilton, Ontario. We assessed all visits with a cannabis-related ICD-10 discharge code 6 months before and after legalization (October 17, 2018) to determine cases of acute cannabis intoxication. The primary outcome was the rate of ED visits. Secondary outcomes included number of visits distributed by age, length of stay, co-ingestions, and clinical course in the emergency department (investigations and treatment).

**Results:**

There was no difference in the overall rate of ED visits following legalization (2.44 vs. 2.94 visits/1000, *p* = 0.27). However, we noted a 56% increase in visits among adults aged 18–29 (*p* = 0.03). Following legalization, a larger portion of patients required observation without interventions (25% vs 48%, *p* < 0.05). Bloodwork and imaging studies decreased (53% vs. 12%, *p* < 0.05; 29% vs. 2%, *p* < 0.05); however, treatment with benzodiazepines increased (24% vs. 51%, *p* < 0.05).

**Conclusions:**

Legalization was not associated with a change in the rate of cannabis-related ED visits in our study. More research is needed regarding changing methods of cannabis ingestion and trends among specific age groups.

## Background

Cannabis has long been used recreationally in various forms: smoked, vapourized, orally ingested or topically applied. Its active ingredients - delta-9-tetrahydrocannabinol (THC) and cannabidiol (CBD) - exert effects on cannabinoid receptors (CB1 and CB2) in the central and peripheral nervous system, along with other organs in the body. THC is considered responsible for many of the plant’s psychiatric and hallucinogenic effects. When consumed in excess, acute cannabis toxicity consists of both psychiatric (euphoria, relaxation, time distortion, loss of inhibitions) and physical effects (tachycardia, conjunctival injection, impairment in cognitive and short-term memory tasks) [1].

The use of cannabis for medicinal purposes, including the treatment of chemotherapy-induced nausea and vomiting, pain, depression, anxiety, insomnia, and tremors due to neurological injury, was legalized in Canada in 2001. By 2016, over 129,000 Canadians were registered users of medical marijuana [2]. Between 2001 and 2016, non-medical cannabis consumption by Canadians grew from an estimated 482 t to 697 t annually [3]. On October 17, 2018, the Cannabis Act decriminalized the recreational use of cannabis. Since legalization, there has been an increase in recreational cannabis consumption, especially among young males, with studies demonstrating an increase in use from 14 to 20% [4]. There has also been an increase in first-time cannabis users across Canada, especially those 45 and older [4]. Inexperienced users may not be aware of the many effects of cannabis, their physiological tolerance, and the delayed-onset of symptoms seen with some routes of ingestion [5].

In other jurisdictions, the legalization of cannabis was associated with increased hospitalizations and emergency department (ED) visits due to cannabis intoxication [6]. For example, Colorado hospitalizations for cannabis intoxication doubled following legalization in 2014 [7]. As such, emergency physicians need to be aware of patterns in cannabis toxicity-related ED visits following legalization. It is also important for public health officials, politicians, and other stakeholders to be aware of the effect legalization has on healthcare utilization. Our study set out to examine the effect of legalization on the rate of ED visits for acute cannabis intoxication in the 6 months before and after the passing of the Cannabis Act at St. Joseph’s Healthcare, an urban academic ED in Hamilton, Ontario, which sees 65,000 ED visits annually and provides psychiatric care for the region**.**

## Methods

### Study design

We conducted a retrospective chart review using data from the Discharge Abstract Database as well as the Epic medical record system at St. Joseph’s Healthcare Hamilton. Our study included all ED visits between April 17, 2018, and April 17, 2019 (6 months preceding and following the Cannabis Act). The study was approved by the Hamilton Integrated Research and Ethics Board at its onset. All visits grouped with a cannabis-related discharge diagnosis were identified, based on the International Statistical Classification of Disease, ICD-10. Specifically, all charts with the code T40.7 (poisoning by cannabis) or F12.* (mental and behavioural disorders due to use of cannabinoids) were identified (see Appendix).

Four abstractors (medical students) were trained on how to use the EMR research platform and a standardized data abstraction form prior to initiating the chart review. Data were extracted by the four medical students as well as by a fifth abstractor (an emergency physician). The emergency physician arbitrated all charts where cannabis intoxication was questionable. The emergency physician also performed a blind review of a random selection of 10% of all charts that were reviewed by the medical students, allowing for the calculation of a kappa statistic for inter-rater reliability for the primary outcome.

Tabulated variables extracted from each chart included: age, sex, date and time of visit, length of stay, chief complaint at triage, synopsis of the visit, co-ingestions or other substances used, tests ordered, medications administered, disposition, and whether the visit was due to acute intoxication from cannabis. The total number of ER visits during this period was obtained.

### Primary and secondary outcomes

Our primary outcome was the rate of ED visits for acute cannabis intoxication before and after legalization. Secondary outcomes compared rate of ED visits distributed by age, length of stay in the emergency department, co-ingestions, and clinical course in the emergency department (investigations, treatment, and ultimate disposition).

### Determining cannabis intoxication

Visits were attributed to acute cannabis intoxication based on predefined criteria: 1) if the ER physician explicitly identified this in the chart or 2) if a consultant or inpatient team identified this in the chart.

### Statistical analysis

Age was described as median with interquartile range, while other continuous variables were presented as mean with standard deviation. All continuous data were compared using independent t-tests. Demographic data, time of visit, chief complaint, co-ingestions, and clinical course in the department were characterized with descriptive statistics and were compared using a chi-squared test.

Rates of cannabis-related visits were calculated per 1000 visits each month over the time interval and compared pre- and post-legalization. Chi-square values and independent t-tests were used to determine the difference between pre- and post-legalization groups. Statistical analyses were performed with the use of Excel and SPSS software. Significance was assessed at the *p* < 0.05 level.

## Results

**Fig. 1:**
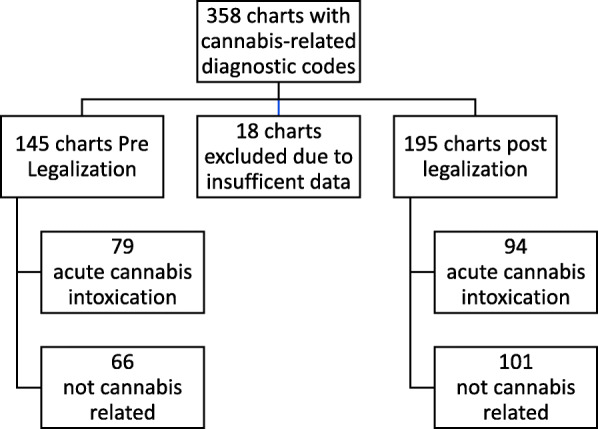
Chart review process

There was a total of 64,152 visits during the 12 months of the study, of which 358 visits had cannabis ICD-10 diagnostic codes. Of these visits, 173 (48%) were attributed to acute cannabis intoxication (Fig. 1), as identified in the chart by the emergency physician or the consultant/inpatient team caring for the patient.

Demographic data are presented in Table 1. The median age of patients was 27, with 68% being male.

**Table 1:** Demographic data of patients presenting to the ED for acute cannabis intoxication

	All (***n*** = 173)	Pre-Cannabis Act (***n*** = 79)	Post-Cannabis Act (***n*** = 94)	***p*** value
**Age (years)** Median (IQR)	27 (22–37)	29 (22.5–39.5)	26 (22–34.5)	0.34
**Age distribution (years)**	18–29	41 (52%)	64 (68%)	0.03
	30–39	19 (24%)	14 (15%)	0.12
	40–49	12 (15%)	8 (9%)	0.17
	50–59	5 (6%)	6 (6%)	0.98
	60 and above	2 (3%)	2 (2%)	0.86
**Sex**				0.14
Male	117 (68%)	58 (73%)	59 (63%)	
Female	56 (32%)	21 (27%)	35 (37%)	
**Rate per 1000 ED visits** Mean (SD)	2.70 (0.74)	2.44 (0.86)	2.94 (0.54)	0.27
**Time of Visit**				0.31
00:00–06:00	32 (18%)	13 (16%)	19 (20%)	
06:01–12:00	26 (15%)	13 (16%)	13 (14%)	
12:01–18:00	60 (35%)	23 (37%)	37 (39%)	
18:01–23:59	55 (32%)	30 (39%)	25 (27%)	
**Chief Complaint**				0.51
-substance abuse	51 (29%)	21 (27%)	30 (32%)	
-physical complaint	42 (24%)	21 (27%)	21 (22%)	
-bizarre behaviour	27 (16%)	14 (18%)	13 (14%)	
-depression	11 (6%)	6 (8%)	5 (5%)	
-hallucinations/delusions	10 (6%)	4 (5%)	6 (6%)	
-anxiety/crisis	7 (4%)	4 (5%)	3 (3%)	
-overdose ingestion	5 (3%)	0 (0%)	5 (5%)	
-other	20 (12%)	9 (11%)	11 (12%)	

Table 2 provides a summary of the clinical course in the emergency department, including length of stay, interventions, co-ingestions, and disposition. The average length of stay for patients that were discharged from the ED was 5.39 h. 32% of patients presented with a co-ingestion, of which the majority was alcohol. 84% of patients were discharged from the emergency department.

**Table 2:** Management and course in the emergency department

	All (***n*** = 173)	Pre-Cannabis Act (***n*** = 79)	Post-Cannabis Act (***n*** = 94)	***p*** value
**Length of Stay in ED, hours**				
Mean (SD)				
Discharged patients	5.39 (4.06)	5.84 (3.89)	5.02 (4.18)	0.23
Admitted patients	27.64 (17.85)	31.49 (23.87)	24.38 (10.51)	0.38
AMA patients	2.39 (0.88)	2.13 (0.47)	2.64 (1.36)	0.70
**Co-ingestions**				
None	118 (68%)	48 (61%)	70 (74%)	0.054
Co-ingestion	55 (32%)	31 (39%)	24 (26%)	
-alcohol		15/31	16/24	0.17
-stimulants (cocaine, meth)		11/31	5/24	0.24
-opioids		7/31	3/24	0.33
-benzodiazepines		2/31	3/24	0.44
**Clinical course in ED**				
Observation	65 (38%)	20 (25%)	45 (48%)	0.002
Intervention	108 (62%)	59 (75%)	49 (52%)	
-bloodwork		31/59	5/49	0.00003
-imaging		17/59	1/49	0.0002
-IV fluids		14/59	8/49	0.34
-benzodiazepines		14/59	22/49	0.02
-antiemetics		16/59	10/49	0.42
-Form 1		5/59	8/49	0.21
-Medicine Consult		1/59	1/49	0.89
-Psychiatry Consult		16/59	10/49	0.42
**Disposition**				0.98
-discharge	145 (84%)	66 (84%)	79 (84%)	
-AMA	4 (2%)	2 (2%)	2 (2%)	
-admit	24 (14%)	11 (14%)	13 (14%)	

### Primary outcome

**Fig. 2:**
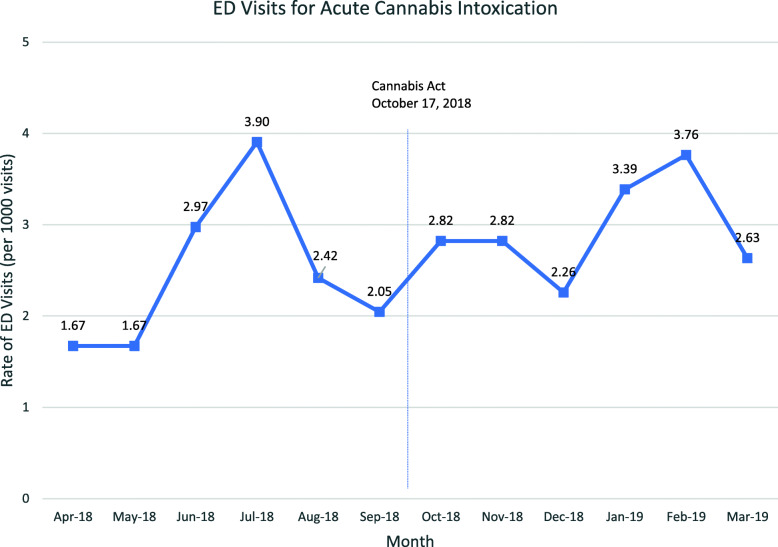
Rate of ED visits for acute cannabis intoxication by month before and after legalization

Figure 2 shows the number of cannabis-related visits per 1000 visits by month during the study interval. There was no difference in overall visits to the ED after legalization (2.44 vs 2.94 visits per 1000 ED visits, *p* = 0.27).

### Secondary outcomes

**Fig. 3:**
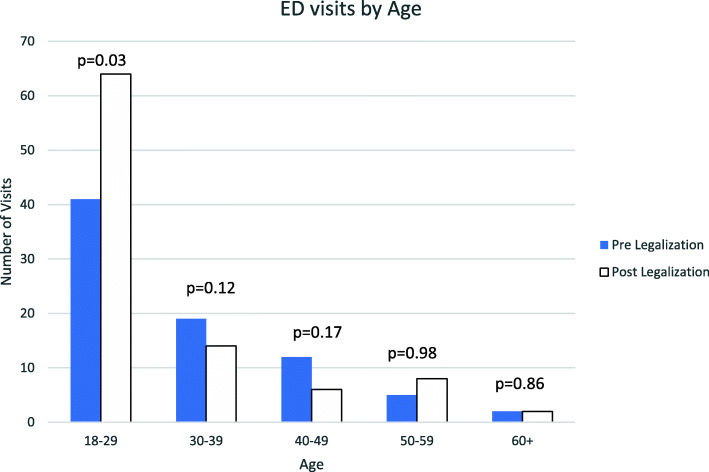
Age distribution of patients presenting for acute cannabis intoxication

Figure 3 shows the distribution of visits by age. The 18–29 age group was associated with the largest increase in visits during the study, a finding that reached statistical significance. After legalization, there was an increase in patients receiving only observation in the department (48% vs 25%, *p* = 0.002), and a reduction in the ordering of bloodwork (53% vs 12%, *p* < 0.05) and imaging (29% vs 2%, *p* < 0.05).

### Inter-rater reliability

There was excellent concordance between reviewers (k = 0.83) in determining cannabis intoxication during the chart review.

## Discussion

To our knowledge, this study constitutes the most extensive examination of documented acute cannabis intoxication ED visits before and after the legalization of recreational cannabis in Canada. Our primary outcome suggests that cannabis legalization was not associated with a change in the rate of ED visits for acute cannabis intoxication.

Patients’ presenting characteristics, including sex and time of visit, remained stable in the 6 months before and after legalization. The median age for all patients was 27, and 62% were male, which is consistent with government data that shows the highest frequency of use in young males [4]. In our analysis of rate of ED visits by age, we found that legalization was associated with a 56% increase (*p* = 0.03) in the number of patients age 18–29 presenting to the ED with acute cannabis intoxication. The management of these patients seemed to change as well. After legalization, more visits consisted of only observation within the ED (48% vs 25%, *p* = 0.002). There was a corresponding decrease in patients requiring bloodwork and imaging. There was no change in length of stay or disposition.

These findings (fewer co-ingestions, decreased investigations, greater tendency towards medical observation) may be partly explained by experimentation by new users after legalization. In this scenario, patients may be seeking medical care as a result of the unpleasant symptoms they are experiencing, which ultimately resolve on their own with time and reassurance [8, 9].

Canadian government data indicates that the largest increase in rates of recreational cannabis use from 2018 to 2019 has been among young males aged 15–24 [4]. Other jurisdictions have also found that young adults and adolescents may be at highest risk of acute cannabis intoxication. Studies from Colorado have identified significant increases in cannabis-related visits for those aged 9–20 following legalization [7, 10, 11]. While this study did not examine patients under the age of 18, we did find an increased rate of ED visits among young adults aged 18–29, which would be consistent with attitudes and usage in this patient population documented by government polls [4]. Furthermore, following legalization in Colorado, studies reported significant increases in mental health consultations among young adults [12]. In contrast, this study found no corresponding increase in the utilization of psychiatric services from the emergency department.

This study is unique in that it used ICD-10-CA codes to capture patient charts with cannabis-related symptoms and then reviewed each chart in detail to identify patients with documented acute cannabis intoxication (Appendix). Since cannabis has a high prevalence of use in the population, charts may be coded with a cannabis-related ICD code when the visit is unrelated to their use. Indeed, we found this to be the case in 52% of the charts we identified. Thus, studies that only interpret trends using ICD codes should be interpreted with caution.

Our single-center study did not include data from the region’s dedicated paediatric emergency department. While we identified significant increases in ED visits among young adults following legalization, our analysis did not capture adolescents under age 18. Additionally, while we identified no change in overall rates of ED visits and differences in the clinical management of ED patients with acute cannabis toxicity, a broader time period of examination may have allowed for a more thorough examination of trends in ED visits, and could also capture seasonal changes in cannabis use and ED visits. Nevertheless, the short interval of our study allowed to closely examine for any sudden changes that may have occurred after legalization.

While we did not examine data based on methods of ingestion, growing concern over edible cannabis and concentrates suggests that future studies should examine patient outcomes based on these parameters [13, 14]. Studies are also needed to investigate similar outcomes while comparing recreational and physician-prescribed cannabis, as current evidence is mostly epidemiological and may not account for dispositional differences [15]. Furthermore, little data is available on the burden that acute cannabis intoxication following legalization has placed on communities of diverse ethnicity, culture, and socioeconomic status, suggesting a need for studies in this field.

The findings in our study also prompt further questions on the utilization of the emergency department by young adults for cannabis intoxication. Reasons for this may include a lower risk perception among younger patients about the adverse effects of cannabis or a lack of knowledge of THC content among consumed products, particularly among first-time users [16, 17]. Further work should be done to determine whether these trends continue in this specific population.

## Conclusions

Following the legalization of cannabis, our study suggests that there was no difference in overall rate of emergency department visits for acute cannabis intoxication. Although patients in this era may require fewer investigations during their ED visit, their length of stay and disposition remain similar compared to the pre-legalization era, indicating mixed effects on ED utilization. Physicians may use this knowledge to predict the management and disposition of ED patients. Future studies should examine these outcomes across broader time periods, as access and attitudes to cannabis evolve.

## Data Availability

The datasets used and/or analysed during the current study are available from the corresponding author on reasonable request.

## Appendix

**Table 3:** ICD-10-CA codes used to query charts using the Discharge Abstract Database at St. Joseph’s Healthcare Hamilton

T40.7	Poisoning by cannabis (derivatives)
**F12.0**	Mental and behavioral disorders due to use of cannabinoids, acute intoxication
**F12.1**	Mental and behavioral disorders due to use of cannabinoids, harmful use
**F12.2**	Mental and behavioral disorders due to use of cannabinoids, dependence syndrome
**F12.3**	Mental and behavioral disorders due to use of cannabinoids, withdrawal state
**F12.4**	Mental and behavioral disorders due to use of cannabinoids, withdrawal state and delirium
**F12.5**	Mental and behavioral disorders due to use of cannabinoids, psychotic disorder
**F12.6**	Mental and behavioral disorders due to use of cannabinoids, amnesic syndrome
**F12.7**	Mental and behavioral disorders due to use of cannabinoids, residual and late-onset psychotic disorder
**F12.8**	Mental and behavioral disorders due to use of cannabinoids, other mental and behavioral disorders
**F12.9**	Mental and behavioral disorders due to use of cannabinoids, unspecified mental and behavioural disorders

## Abbreviations

ED: Emergency department
THC: Delta-9-tetrahydrocannabinol
CBD: Cannabidiol
ICD: International Statistical Classification of Disease

## References

[1] Borgelt LM, Franson KL, Nussbaum AM, Wang GS (2013). The pharmacologic and clinical effects of medical cannabis. Pharmacotherapy..

[2] Government of Canada SC (2018). Licensed cannabis industry statistics.

[3] Macdonald R, Rotermann M (2017). Experimental estimates of cannabis consumption in Canada, 1960 to 2015. Economic Analysis Division and Health Analysis Division, Statistics Canada.

[4] Statistics Canada (2019). National Cannabis Survey, first quarter 2019.

[5] Ashton CH (2001). Pharmacology and effects of cannabis: a brief review. Br J Psychiatry.

[6] Yeung M, Janz K, Weaver C, Saah-Haines R, Lang E (2019). Cannabis in the emergency department: the impact of cannabis legalization on cannabis and opioid-related presentations. Alberta Acad Rev.

[7] Kim HS, Monte AA (2016). Colorado cannabis legalization and its effect on emergency care. Ann Emerg Med.

[8] Agaku I, Omaduvie U, Vardavas C, Filippidis F. Cannabis use is associated with reduced harm perception towards illicit drugs and experimentation with new psychoactive substances among European adolescents and young adults. Eur Respir J. 2015;46:PA5118. 10.1183/13993003.congress-2015.PA5118.

[9] Danseco ER, Kingery PM, Coggeshall MB (1999). Perceived risk of harm from marijuana use among youth in the USA. Sch Psychol Int.

[10] Wang GS, Roosevelt G, Heard K (2013). Pediatric marijuana exposures in a medical marijuana state. JAMA Pediatr.

[11] Mechcatie E (2018). The impact of legalization of medical and recreational marijuana. AJN Am J Nurs.

[12] Roberts BA (2019). Legalized cannabis in Colorado emergency departments: a cautionary review of negative health and safety effects. West J Emerg Med.

[13] Brown S (2019). Emergency physicians and public health experts call for tight regulations on cannabis edibles and concentrates. CMAJ..

[14] Zipursky JS, Bogler OD, Stall NM (2020). Five things to know about edible cannabis. CMAJ..

[15] Woodruff SI, Shillington AM (2016). Sociodemographic and drug use severity differences between medical marijuana users and non-medical users visiting the emergency department. Am J Addict.

[16] Yeomans-Maldonado G, Patrick ME (2015). The effect of perceived risk on the combined used of alcohol and marijuana: results from daily surveys. Addict Behav Rep.

[17] Volkow ND, Baler RD, Compton WM, Weiss SRB (2014). Adverse health effects of marijuana use. N Engl J Med.

